# Characterization of Foot and Mouth Disease Virus Serotype SAT-2 in Swamp Water Buffaloes (*Bubalus bubalis*) under the Egyptian Smallholder Production System

**DOI:** 10.3390/ani11061697

**Published:** 2021-06-07

**Authors:** Hend M. El Damaty, Elshaima M. Fawzi, Ahmed N. F. Neamat-Allah, Ibrahim Elsohaby, Abdelmonem Abdallah, Gamelat K. Farag, Yousry A. El-Shazly, Yasser S. Mahmmod

**Affiliations:** 1 Infectious Diseases, Department of Animal Medicine, Faculty of Veterinary Medicine, Zagazig University, Zagazig City 44511, Egypt; hendvet11@yahoo.com (H.M.E.D.); elshaimafawzi@yahoo.es (E.M.F.); yasserpcr@gmail.com (Y.S.M.); 2 Department of Clinical Pathology, Faculty of Veterinary Medicine, Zagazig University, Zagazig City 44511, Egypt; anattia@vet.zu.edu.eg; 3 Department of Health Management, Atlantic Veterinary College, University of Prince Edward Island, Charlottetown, PE C1A4P3, Canada; 4 Internal Medicine, Department of Animal Medicine, Faculty of Veterinary Medicine, Zagazig University, Zagazig City 44511, Egypt; abd.el.monem.ali@umontreal.ca; 5 Department of Virology, Faculty of Veterinary Medicine, Zagazig University, Zagazig City 44511, Egypt; sendooo2002@gmail.com; 6 Faculty of Veterinary Medicine, Zagazig University, Veterinary Hospital, Zagazig City 44511, Egypt; yousryabdelfatah229@gmail.com; 7 Division of Veterinary Sciences, Faculty of Health Sciences, Higher Colleges of Technology, Al Ain 17155, United Arab Emirates

**Keywords:** foot and mouth disease, water buffalo, Egypt, serotype SAT-2, VP sequencing, FMD outbreak, cardiac marker

## Abstract

**Simple Summary:**

Continuous mutations of the foot and mouth disease virus (FMDV) result in the emergence of new topotypes and lineages of FMDV, which contribute to an occasional vaccination failure. Thus, molecular characterization of the circulating FMDV strains associated with a recent outbreak in Egyptian water buffaloes is essential for the control strategies. The results revealed that the circulating virus was SAT-2 serotype, closely related to the lineage of lib12, topotype VII, with a similarity of 98.9%. The new incursions reported in this study explain the considerable high morbidity of FMDV outbreaks in Egypt in early 2019.

**Abstract:**

Spontaneous mutations are a common characteristic of the foot and mouth disease virus (FMDV), leading to wide antigenic variations resulting in the emergence of new topotypes and lineages of FMDV, which contributes to occasional vaccination failures. The objectives of the present study were to genetically characterize FMDV isolated from water buffaloes and study the biochemical and histopathological indicators of infected animals. Fifty-four water buffaloes of both sexes and different ages suffered from acute symptoms of FMD were clinically examined and randomly selected for inclusion in this study. Oral desquamated epithelial and oropharyngeal fluid samples have been tested for FMDV by reverse transcriptase PCR (RT-PCR). Tissue and serum samples were also collected from the diseased buffaloes and subjected to histopathological and biochemical analysis. Our findings showed that all examined samples were confirmed to be positive to FMDV serotype SAT-2 and were adjusted to be responsible for the recent disease outbreak in this study. Phylogenetic analysis revealed that the circulating viruses were of the SAT-2 serotype, closely related to the lineage of lib12, topotype VII, with 98.9% identity. The new lineage of SAT-2 showed a high virulence resulting in the deaths of water buffaloes due to heart failure, confirmed by high serum levels of inflammatory and cardiac markers, including haptoglobin, ceruloplasmin, cardiac troponin I and creatine phosphokinase-MB, indicating an unfavorable FMD-infection prognosis. In conclusion, we document the presence of new incursions circulating in water buffalo populations in Egypt in early 2019, explaining the high morbidity rate of FMD outbreak in early 2019. Furthermore, the newly identified serotype SAT-2 lib12 lineage, topotype VII, showed an aggressive pattern in water buffaloes of the smallholder production system.

## 1. Introduction

Foot and mouth disease (FMD) is a devastating, highly contagious viral disease of all cloven-hoofed animals caused by the foot and mouth disease virus (FMDV) of the *Picornaviridae* family. The disease poses a severe threat globally, impeding the international trade of live animals and animal by-products [1,2]. FMDV includes seven serotypes, namely, A, O, C, Southern African Territories (SAT)-1, SAT-2, SAT-3, and Asia1. Each serotype has antigenically distinct subtypes due to the high mutation rate [3].

FMD is endemic in many countries in Africa, Asia, and South America [4,5]. In Egypt, FMDV serotypes O and A are endemic and cause sporadic outbreaks [6]. During 2012, widespread outbreaks due to FMDV serotype SAT-2 were reported in the Delta and Upper Egypt Governorates [7]. Since 2012, livestock in Egypt have suffered occasional FMD outbreaks due to different FMDV strains, resulting in significant losses among calves [8,9]; moreover, even co-infections with other infectious pathogens have been reported [10]. The appearance of new lineages from the different serotypes is associated with increased mortality rates in young ruminants and adults, inflicting severe economic losses [11,12]. All FMDV serotypes produce a clinically indistinguishable disease, but immunity to one serotype does not protect against the others due to the wide antigenic diversity. The affected livestock commonly exhibits fever; the cessation of rumination; ruby salivation; and the eruption of blisters on the lips, tongue, mouth, nose, between the toes, and sometimes on the teats; along with decreased milk production [9,13].

Genetic analysis of viral protein 1 (VP1-coding region), the most variable protein among structural capsid polypeptides, can classify each serotype’s strains into topotypes, lineages, and genotypes essential in tracing the cause of the newly emerging strains [14]. Serotype SAT-2 strains were clustered into 14 topotypes (I–XIV) by phylogenetic analyses. Topotype VII is the only one categorized in Egypt, which is predominantly endemic in south Sub-Saharan countries [10,15].

Domestic Nile water buffaloes (*Bubalus bubalis*) are essential animals for most farmers in Egypt, given their multiple advantages over cattle. In particular, water buffaloes are disease-resistant, produce high-fat milk and high-quality meat, and adapt to different environments with distinct climates. Egyptian farmers use water buffaloes for many purposes, including drafting animals and assisting with crop work in the fields. Numerous studies have reported and described FMD in cattle and other ruminant populations [10,16,17]. However, there has been little research on FMD in domestic water buffaloes.

The smallholder production system by traditional householders is a common livestock production system in Egypt, which is scattered throughout the country [18]. This traditional breeding system is typically used for small herds or small numbers of animals kept for subsistence or as a source of additional income by households. However, most studies on FMD have focused on farms with intensive production systems, which follow standard routine management, vaccination programs, and control measures against infectious diseases, including FMD [7,12,16]. Furthermore, variations in the microenvironment and management practices between these two production systems could influence the epidemiology and endemicity of FMD.

In early 2019, a massive outbreak of FMD hit livestock across Egypt, particularly in the Delta region. Thus, the objectives of this study were to (1) genetically characterize the circulating FMDV associated with the recent outbreak in the Sharkia and Dakahlia Governorates, Egypt, during the first quarter of 2019, and (2) study the histopathological profile and biochemical indicators in infected water buffaloes (*Bubalus bubalis*) to obtain a complete picture of the virulence of the circulating serotype.

## 2. Materials and Methods

### 2.1. Study Population and Sampling

In the first quarter of 2019, 54 water buffaloes aged from 2 months to 8 years, including 40 females and 14 males, were randomly selected for sampling and inclusion in this study. Buffaloes were clinically examined following the process described by Constable et al. [19]. Animals showing typical clinical signs, including blister formation on the mouth and feet, high fever, lameness, and inappetence, were selected. The selected buffaloes were raised by smallholders under a semi-intensive program in different Sharkia and Dakahlia Governorates villages and were vaccinated with Tri-Aphthovac FMDV vaccine (Middle East Company for Veterinary Vaccine (MEVAC), Sharkia, Egypt).

Oropharyngeal swabs and oral desquamated epithelia were collected from the clinically FMD-infected buffaloes. Such samples were kept in a transportation medium containing equivalent amounts of glycerol, phosphate-buffered saline (pH 7.2–7.6), and 2% antibiotic/antimycotic (BioWhittaker, Walkersville, MD, USA). In addition, whole blood was collected into sterile 10-mL vacuum tubes without anticoagulant by jugular venipuncture. Tissue samples from the ventricular parts of the heart were also collected during the post-mortem examination of seven newly deceased buffaloes with heart lesions. All samples were labelled and transported on ice to the laboratory for later analysis.

### 2.2. Nucleic Acid Extraction and RT-PCR

Whole nucleic acid extraction from the oropharyngeal swabs was performed using the QIAamp MiniElute Virus Kit (Qiagen, Hilden, Germany), following the manufacturer’s recommendations. The oligonucleotide primer pairs (Metabion, Germany) used in the reverse-transcription PCR (RT-PCR) are listed in Table 1. In a PTC-100 TM programmable thermal cycler (MJ Research Inc., Waltham, MA, USA), the PCR amplification reactions were conducted with a reaction mixture with a final volume of 25 μL as follows: 12.5 µL of Quantitect probe buffer RT-PCR (Qiagen-GmbH), 1 µL of each primer (at a concentration of 20 pmol), 4.25 µL of water, 0.25 µL of RT-enzyme, and 6 µL of a template. Reverse transcription was applied at 50 °C for 30 min; a primary denaturation step was performed at 95 °C for 5 min, followed by 35 cycles of 94 °C for 30 s, 55 °C for 30 s, and 72 °C for 30 s. For FMD A and O, annealing was performed at 55 °C for 40 s, while for SAT-2, it was performed at 60 °C for 40 s. The final extension phase was performed at 72 °C for 12 min. The PCR products were run on a 1% agarose gel with negative and positive controls (O/EGY/3/93 (EU553840); A/EGY/1/2006 (EF208757); SAT-2/EGY/9/2012 (JX014255)) of FMD serotypes. Controls were kindly provided from the Biotechnology Unit, Animal Health Research Institute, Dokki, Giza, Egypt.

### 2.3. DNA Sequencing and Phylogenetic Analysis

Representative samples of the FMD cases (*n* = 5) were subjected to DNA sequencing. SAT-2 amplicons were purified using a QIAquick PCR Product Extraction Kit (Qiagen, Valencia, CA, USA). Bigdye Terminator V3.1 cycle sequencing kit (Perkin-Elmer, Waltham, MA, USA) was used for the sequence reaction, followed by purification using Centrisep spin columns. DNA sequences were obtained using Applied Biosystems 3130 genetic analyzer (Hitachi, Tokyo, Japan). A BLAST^®^ analysis (Basic Local Alignment Search Tool) [23] was initially performed to establish sequence identity to GenBank accessions. The VP1-coding sequences from the present study were deposited in GenBank under accession numbers MN864514–MN864518. The nucleotide sequences were aligned with other VP1-coding sequences at GenBank after performing a Blast search. The maximum likelihood method was used to construct the phylogenetic tree through MEGA X software [24]. Phylogenetic relationships were estimated using 1000 bootstrap repeats. The evolutionary distances were computed using the P-distance method.

### 2.4. Biochemical Analysis

Whole blood samples collected from the FMD-infected buffaloes (*n* = 54) were centrifuged at 1500× *g* for 10 min at room temperature to separate serum for assessing cardiac troponin I (cTnI), creatine phosphokinase-MB (CK-MB), haptoglobin, and ceruloplasmin. The serum cTnI reactivity was measured using commercially available ELISA kits (CARD-I-KIT ELISA cTnI, Labmaster Oy, Turku, Finland) following manufacturer instructions. However, CK-MB, haptoglobin, and ceruloplasmin were measured using commercially available kits purchased from Bio-diagnostic Kits, Giza, Egypt. Analysis was performed as described previously [25,26,27].

### 2.5. Histopathological Examination

Tissue samples from the heart were fixed at 10% neutral formalin solution buffer for 18–24 h. Samples were trimmed, washed, dehydrated and then embedded in paraffin wax. Five micron paraffin-embedded sections were primed and stained with hematoxylin and eosin and examined under a light microscope [28].

### 2.6. Data Analysis

The PC-ORD Software (v. 5; Gleneden Beach, OR, USA), the web-based tools MetaboAnalyst [29] and Heatmapper [30], and SPSS software (v. 25; IBM, Armonk, NY, USA) were used for bioinformatics and statistical analyses. All values are presented as mean ± standard error (SE). Results are considered significantly different at *p* ≤ 0.05.

## 3. Results

**Figure 1:**
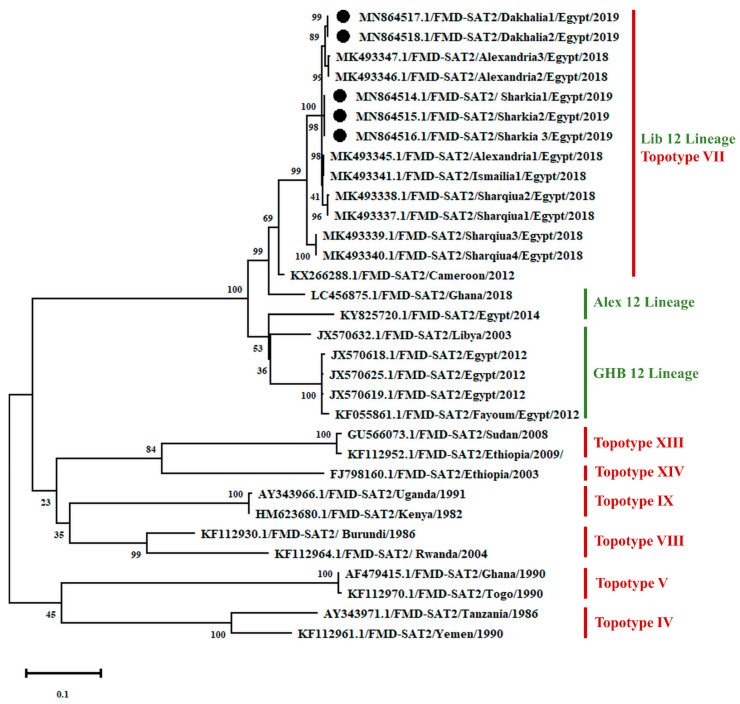
Phylogenetic analysis of the VP1-coding sequences showing the relation between different FMD SAT-2 of various topotypes. The tree was constructed using the Hasegawa–Kishino–Yano model as a maximum likelihood method and a discrete Gamma distribution (+G) with five rate categories and assuming that a certain fraction of sites are evolutionarily invariable (+I) at 1000 bootstraps. The black colour circles refer to the FMD isolates recovered in the present study.

Of all tested samples (*n* = 54), RT-PCR assay amplified cDNA fragments using the 5′UTR universal primers with the expected 326 bp amplicon and confirmed FMDV being responsible for the disease symptoms that clinically affected water buffaloes. All samples tested positive for SAT-2 but negative for A and O serotypes using primers specific for each serotype. VP1-coding sequencing and phylogenetic analysis revealed a substantial degree of relatedness between the representative Egyptian isolates (*n* = 5) with a similarity of 98.9, which clustering in topotype VII, lib12 lineage (Figure 1). This confirmed that the SAT-2 serotype was responsible for the FMD outbreak in early 2019 in the Sharkia and Dakahlia Governorates, Egypt.

**Figure 2:**
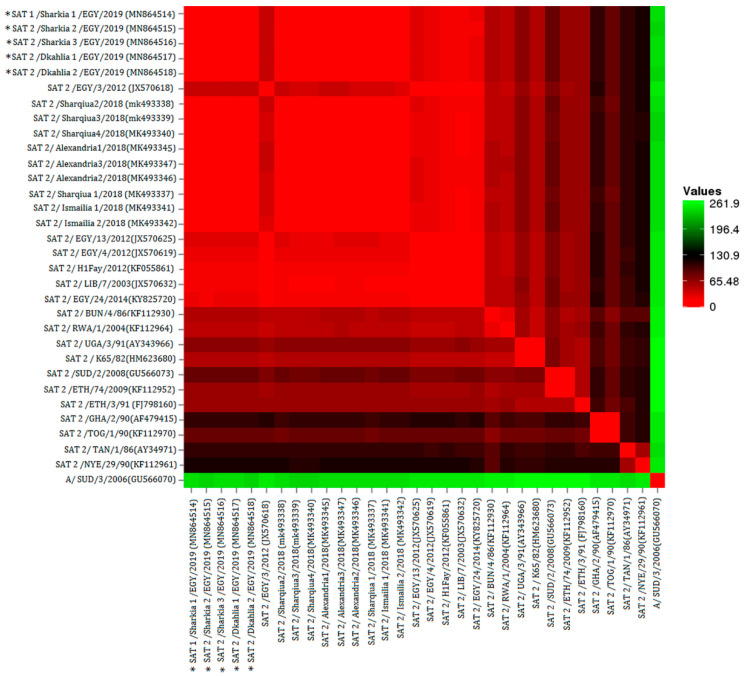
The pairwise distance among the studied FMD isolates. The Euclidean distance was calculated based on the VP1-coding sequences and is shown as colours. The colour key represents the Euclidean distance. The (*) refers to the FMD isolates recovered in the present study.

Furthermore, phylogenetic analysis and pairwise comparison of the VP1-coding sequences revealed high similarity between the five SAT-2 isolates sequenced in the present study and other strains circulating in Egypt [SAT-2/Alexandria2/2018 (MK493346), SAT-2/Sharqiua2/2018 (MK493338), and SAT-2/Ismailia1/2018 (MK493341)] (Figure 1 and Figure 2). However, it showed a 12.2–12.7% variance from the previously isolated strain in Gharbia 2012 (SAT-2 EGY/3/2012 topotype VII, lineage Gharbia 12; JX570618), also our strains differed from the previously isolated strain in Alexandria 2014 (KY825720) with variance 15.1–18.7%.

**Figure 3:**
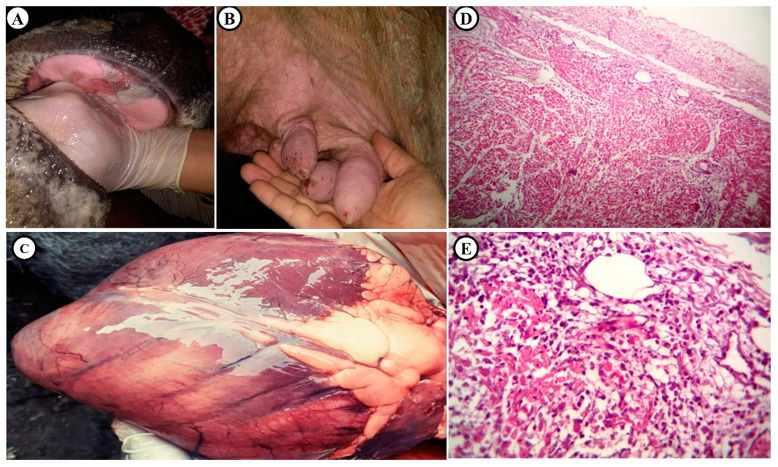
Clinical and histopathological signs in FMD-infected buffaloes. (**A**) Blisters and ulcers in the dental pad. (**B**) Blisters in teats. (**C**) Heart of buffalo showing grayish-white stripping, particularly in the left ventricle near the septum (Tiger heart). (**D**) Heart of buffaloes showing pericarditis and myocarditis ×120 H&E; and (**E**) Heart of buffaloes showing non-suppurative myocarditis characterized by replacement of necrotic cardiac myocytes by edema, lymphocytes, and some plasma cells ×400 H&E.

Infected buffaloes showed typical FMD clinical signs, including frothing of mouth and ropy salivation, a vesicular eruption of the buccal mucosal cavity, the skin of udder and teat orifices (Figure 3A,B). Cardiac auscultation detected the muffled, frictional, and tinkling-splashing sounds. The estimated serum levels of cTnI (0.61 ± 0.02 µg/L), CK-MB (263.73 ± 5.56 U/L), haptoglobin (0.493 ± 0.051 g/dL), and ceruloplasmin (17.72 ± 1.23 mg/dL) in clinically FMD-infected buffaloes were significantly higher than the standard ranges (Table 2).

The FMD-affected hearts from the clinical cases showed pathogenic lesions typical of FMD in the post-mortem and histopathological examinations, in the form of pericarditis, represented by a grayish-white edematous membrane and thickened pericardium. The myocardium, especially in the left ventricle near the septum, showed grayish-white stripping (Figure 3C). Microscopically, there was serofibrinous pericarditis with varying degrees of non-suppurative myocarditis involving sub-pericardium muscles, as well as inflammatory edema around the inter-muscular blood vessels (Figure 3D). The affected myocardial muscles showed various degrees of degeneration and necrosis, with partial replacement by edema, intense inflammatory cells (mainly lymphocytes), and some plasma cells besides mild fibroblastic proliferation, reflecting the presence of non-suppurative myocarditis and pericarditis (Figure 3E).

## 4. Discussion

Various smallholders/householders from villages in Sharkia and Dakahlia Governorates, Egypt, reported clinical signs of FMD among vaccinated water buffaloes (*Bubalus bubalis*) in the first quarter of 2019. The present study aimed to genetically characterize the circulating FMDV virus strains associated with this outbreak. Furthermore, we assessed the virulence of the circulating serotype by examining the histopathological profile and biochemical indicators in FMD-infected buffaloes.

Our findings revealed that the SAT-2 serotype was responsible for the FMD outbreak in early 2019 in Egypt. This finding is consistent with an alert on the 2018 FMD outbreak [12], which reported the emergence of the FMDV Lib-12 lineage of topotype VII, serotype SAT-2, in Egypt. There are three predominant FMDV serotypes in Egypt: O, A, and SAT-2 [35,36,37]. However, in recent decades, several emerging topotypes, lineages, and genotypes within these three well-established serotypes have been documented in many reports [6,12,16,35].

Our sequenced strains from the 2019 outbreak isolates (MN864514–MN864518) showed close relatedness to Ghanaian strains reported in the 2018 outbreak (LC456875) with an identity 91.10% and West and Central Africa strains reported in 2016 (KX266288) with an identity 94.14%. However, the SAT-2 serotype isolated in the present study showed less similarity to the SAT-2 serotype reported in Ethiopia during 2009 outbreaks (KF112952) with 72.51% similarity, the SAT-2 serotype identified in Sudan in 2008 (GU566073) with 72.51% similarity and the SAT-2 serotype isolated in Uganda in 2009 (FJ461346), with an identity 79.59%. This finding suggested that the new SAT-2 serotype lineage circulating in Egypt in 2019 might have been introduced to Egypt from Ghana and/or West and Central Africa, possibly through importing animals and animals’ by-products. On the contrary, Ahmed et al. [6] reported that serotype SAT-2/VII/Ghb-12 (JX570618) had emerged in Gharbia Governorate, Egypt, during 2012 and caused devastating losses to cattle. Furthermore, Soltan et al. [12] detected SAT-2 Alx-12 lineage of topotype VII (SAT-2/Fayoum1/Egy/2017; MF322696) in nonvaccinated cattle in Dakahlia and Fayoum Governorates, Egypt, between 2016 and 2017.

The phylogenetic tree revealed a substantial degree of relatedness between the representative Egyptian isolates (*n* = 5) with a similarity of 98.9%, although these isolates were recovered from two different districts of Egypt. However, it showed a 12.2–12.7% variance from the vaccine strain registered under accession number (JX570618); namely, FMDV SAT-2 EGY/3/2012 topotype VII, lineage Gharbia 12. The variation between the isolated and vaccine strains explains the widespread and aggressiveness of the FMDV involved in the new outbreak in early 2019. Moreover, this clarifies the markedly high morbidity in livestock and the high mortality, particularly among young calves, despite the annual vaccination program.

After the end of this study, the Egyptian Veterinary Authority responded to the farmers’ warnings about the early 2019 FMD outbreak via an emergency vaccination campaign using the regularly used trivalent vaccines. However, our findings show that this strategy was inadequate to provide complete protection based on the sequence data and phylogenetic analysis. A similar conclusion was previously reported [12] and suggested the manufacture of local monovalent vaccines containing this recent strain (SAT-2 serotype, topotype VII, lib12 lineage).

FMD is known to cause high mortality rates (sometimes 100%) among calves younger than 2 months due to FMDV tropism switching to the cardiac muscle, causing myocarditis [38,39]. In the present study, the mortality rate among adult buffaloes aged more than 2 years was unexpectedly as high as 14/54 (25.9%). A previous study suggested using cardiac biomarkers such as cTnI to assess the prognosis of FMD [40]. The cardiac biomarkers from clinically FMD-infected buffaloes were highly elevated compared with the standard reference ranges. This reflects the aggressiveness and high virulence of the circulating FMD serotype, which was also confirmed by histopathological analysis of the newly deceased buffaloes’ hearts and elevated acute-phase proteins such as haptoglobin and ceruloplasmin [41,42]. However, we think these findings were exacerbated by complications associated with other factors, such as emaciation due to parasitic infections and malnutrition of the livestock raised under the smallholder production system [18].

In addition, farmers raising animals under the smallholder production system tend to overlook the registration of newborn calves, either to avoid paying extra taxes or to sell them to local merchants for extra income (personal communications). Thus, these animals are not registered and subsequently are not listed for the annual FMD vaccination program. Therefore, they lack immunity against FMD and are particularly susceptible to FMDV [43]. Unlike large-scale animal production farms, the hygiene and biosecurity measures in smallholder farms are insufficient. Therefore, the disease spread and the consequences of this among smallholder farmers are devastating.

## 5. Conclusions

This study documented new incursions circulating in water buffalo populations in Egypt in early 2019, which explains the high morbidity rate of FMD outbreak in early 2019. The newly identified serotype SAT-2 lib12 lineage, topotype VII showed an aggressive pattern in water buffaloes of the smallholder production system. Implementing strict control measures against FMD under a smallholder production system is crucial, and the inclusion of the new serotype SAT-2 lib12 lineage, topotype VII, is important for efficient vaccination and its subsequent protection.

## Figures and Tables

**Table 1:** Primer sequences, target genes, and amplicon sizes, used for characterizing foot and mouth disease virus (FMDV).

Serotype	Target Genes	Primer Sequence (5′–3′)	Length of Amplified Product (bp)	Reference
FMD	5′UTR	GCCTGGTCTTTCCAGGTCT	326	[20]
		CCAGTCCCCTTCTCAGATC		
FMD A	VP3-2B	TACCAAATTACACACGGGAA	863–866	[21]
		GACATGTCCTCCTGCATCTG		
FMD O	VP3-2B	ACCAACCTCCTTGATGTGGCT	1301	
		GACATGTCCTCCTGCATCTG		
FMD SAT-2	VP3-2B	TGAACTACCACTTCATGTACACAG	1279	[22]
		ACAGCGGCCATGCACGACAG		

**Table 2:** The standard range and mean concentrations of the cardiac troponin I (cTnI), creatine phosphokinase-MB (CK-MB), haptoglobin, and ceruloplasmin in 54 FMD-infected buffaloes.

Parameters	Standard Concentration Ranges	Mean (±SE)	References
cTnI (µg/L)	0.021–0.12	0.61 ± 0.02	[31,32]
CK-MB (U/L)	100.56–165.64	263.73± 5.56	[31]
Haptoglobin (g/dL)	0.191–0.260	0.493 ± 0.051	[33]
Ceruloplasmin (mg/dL)	8.65–10.86	17.72 ± 1.23	[34]

## Data Availability

The data presented in this study are available on request from the corresponding author.
